# S200. HYPOVITAMINOSIS D IN SCHIZOPHRENIA: PREVALENCE AND ASSOCIATED CLINICAL CHARACTERISTICS

**DOI:** 10.1093/schbul/sby018.987

**Published:** 2018-04-01

**Authors:** Rahma Nefzi, Amine Larnaout, Hanen Ben Ammar, Emira Khelifa, Amina Aissa, Zouhaier El Hechmi

**Affiliations:** Razi Hospital

## Abstract

**Background:**

Schizophrenia is an invalid and severe neurodevelopmental disorder. The implication of vitamin D in the etiopathogenesis of schizophrenia shows through the activation of cellular and inflammatory pathways. It is especially vitamin D deficiency that has been associated with schizophrenia.

It is within this framework that this study aims to explore the relationship between vitamin D levels and the clinical characteristics in a cohort of Tunisian patients with schizophrenia.

**Methods:**

A cross-sectional and retrospective descriptive study was conducted at the “F” psychiatry department at the Razi Hospital, Manouba over a twelve-month period from June 1st, 2015 to May 31st, 2016, including 80 patients with schizophrenia in period of clinical remission. The evaluation focused on sociodemographic and clinical characteristics. A dosage of vitamin D was performed.

**Results:**

The patients had an average age of 42.5 years and 70% were male. The average vitamine D level was 10,57ng/ml ±5,9. 49% of patients had vitamin D insufficiency (between 10 and 30 ng/ml) and 51% had vitamin D deficiency (<10 ng/ml). Vitamin D levels had not been affected by the clinical characteristics of the disease. A negative correlation with the total score of the negative scale (p <0.001) as well as with the severity item of the clinical global impression scale (p = 0.01) were found.

**Discussion:**

A large number of research studies in immunogenetics and molecular biology have highlighted the involvement of vitamin D in the etiopathogenesis of schizophrenia through its role in the ontogenesis of dopaminergic systems and also through its intervention in the processes of neuro-protection, immunomodulation and the reduction of oxidative stress.

In addition, it has been established that people with psychotic disorders have a high prevalence of vitamin D deficiency, but the correlates and relevance of this deficiency remain unclear.

